# Carbohydrate composition of infant formula and glycemic regulation in early infancy using continuous glucose monitoring: cross-sectional evidence of altered glucose patterns with corn syrup solid-based formulas

**DOI:** 10.1016/j.ajcnut.2026.101325

**Published:** 2026-05-19

**Authors:** Sevan Esaian, Beth A Smith, Jinseok Oh, Juan C Espinoza, Alaina P Vidmar, Michael I Goran

**Affiliations:** 1 Department of Pediatrics, Children’s Hospital Los Angeles, Los Angeles, CA, United States; 2 Developmental Neuroscience and Neurogenetics Program, The Saban Research Institute, Children’s Hospital Los Angeles, Los Angeles, CA, United States; 3 Division of Developmental-Behavioral Pediatrics, Children’s Hospital Los Angeles, Los Angeles, CA, United States; 4 Department of Pediatrics, Keck School of Medicine, University of Southern California, Los Angeles, CA, United States; 5 Consortium for Technology & Innovation in Pediatrics, Ann & Robert H. Lurie Children’s Hospital of Chicago, Chicago, IL, United States

**Keywords:** human milk, continuous glucose monitor, corn syrup solids, glucotype, glycemic variability, infant, infant formula, lactose, type 2 diabetes

## Abstract

**Background:**

Infant formulas vary widely in carbohydrate composition, yet associations between exposure to nonlactose carbohydrates and glycemic patterns in early infancy remain poorly characterized.

**Objectives:**

We assessed associations between infant feeding strategy and continuous glucose monitor (CGM)-derived measures of glycemic variability in a cross-sectional observational cohort of infants at 6 mo of age.

**Methods:**

Forty-five infants (28.0 ± 1.2 wk; 47% female) wore CGMs recording interstitial glucose every 15 min for 3 to 8 d. Feeding strategy was categorized as exclusive human milk, formula containing lactose or corn syrup solids (CSS), or mixed human milk/lactose-based formula. Twenty-eight CGM-derived metrics were computed using the R package *iglu*. Group differences were tested using Freedman–Lane analysis of covariance with permutation-based post hoc tests; effect sizes (_η_^2^_p_) and 95% bootstrap confidence intervals (BCI) were reported for all key comparisons. Exploratory hierarchical clustering (Ward’s D2) examined glycemic variability subgroups independent of feeding strategy.

**Results:**

Approximately 46% of CGM-derived metrics differed significantly across feeding strategies, all reflecting contrasts between CSS-based formula and other groups; no metrics differed among human milk, lactose-based formula, or mixed feeding. Compared with human milk, CSS-fed infants were associated with greater glycemic variability and large effect sizes (though the study was powered only to detect large effects), including greater time in hyperglycemia (_η_^2^ = 0.21; 95% BCI = − 2.59,2.49), glycemic risk assessment diabetes equation (_η_^2^ = 0.31; 95% BCI = − 0.25,0.24), J index (_η_^2^ = 0.24; 95% BCI = − 1.07,1.08), and mean amplitude of glycemic excursions (_η_^2^ = 0.40; 95% BCI = − 6.14,6.03). Exploratory clustering identified 4 glycemic variability subgroups. One subgroup exhibited broadly elevated glucose variability and included ~36% of CSS-fed infants, with no representation from other feeding strategies.

**Conclusions:**

Infant feeding strategy is associated with differences in CGM-derived glycemic variability at 6 mo, driven by greater glucose variability among CSS-fed infants. Human milk and lactose-based formula feeding do not differ. Exploratory analyses identify a subgroup with pronounced glycemic variability that includes a subset of CSS-fed infants, highlighting interindividual variability in glycemic response.

## Introduction

In the United States, infant feeding exposures vary substantially because of heterogeneity in infant formula composition. The 1980 Infant Formula Act (21 CFR 107.100) [1–3] established minimum nutrient requirements but did not specify carbohydrate sources or upper limits, requiring only that ingredients be “generally recognized as safe” [3,4]. Therefore, formula products increasingly incorporate nonlactose carbohydrates [5–8]. More than half of infant formula sales are now lactose-reduced, and formulas containing corn syrup solids (CSS) have increased markedly in prevalence according to NHANES data from 1999 to 2020 [9]. In contrast, lactose remains the only digestible carbohydrate in human milk [10]. These shifts underscore the importance of understanding whether differing carbohydrate profiles are associated with measurable differences in infant metabolic physiology.

Randomized trials comparing lactose-based and lactose-reduced formulas in healthy infants demonstrate comparable growth outcomes but have not evaluated longer-term behavioral or metabolic endpoints [11–13]. Emerging observational studies suggest that lactose-reduced formulas may be associated with differences in behavior, metabolism, and obesity risk [14,15]. Previous work found that formulas containing CSS predicted sharper increases in food fussiness and lower enjoyment of food between 12 and 24 mo, disruptions to gut–microbiome development at 6 mo, and higher toddler intake of sugar-sweetened beverages [15,16]. In a cohort of over 15, 000 infants enrolled in California’s Women, Infants & Children Program, CSS-based formula feeding was also associated with a 10% higher risk of obesity and greater consumption of sugary beverages at ages 2 and 4, with evidence of a dose–response relationship [13,17].

One potential pathway linking formula carbohydrate composition to later outcomes is acute glycemic patterning. CSS-based formulas have a higher glycemic index than human milk or lactose-based formulas [14]. Whereas lactose consists of glucose and galactose, CSS are predominantly glucose polymers, a carbohydrate source with a higher glycemic index than lactose [12,14]. Infant formulas with CSS therefore provide a greater proportion of readily absorbable glucose relative to lactose-based formulas, which has been hypothesized to influence glycemic and metabolic responses [18]. Controlled feeding studies in infants have demonstrated distinct postprandial metabolic profiles among those fed CSS-based formula compared with lactose-based formula or human milk, including differences in glucose and insulin dynamics over the postfeeding period [12,18]. However, few studies have characterized glucose dynamics across daily feeding contexts, in part because repeated blood sampling in infants is challenging [19–21]. Continuous glucose monitors (CGM) provide a minimally invasive method to assess real-world glycemic patterns during infancy [22,23].

Our primary aim was to evaluate associations between infant feeding strategy and CGM-derived glycemic patterns at 6 mo of age among infants exclusively fed human milk, formula containing lactose compared with CSS, or mixed (human milk/lactose formula). Given the observational design, we focused on describing differences in glucose variability and temporal patterns. We also used hierarchical clustering to identify additional glycemic patterns not captured by predefined feeding strategy comparisons.

## Methods

All study procedures were approved by Children’s Hospital Los Angeles’ Institutional Review Board (IRB) (CHLA-22–00097). Both parents/legal guardians signed a written informed consent form before the participation of the mother/infant dyad (unless 1 was deceased, unknown, incompetent, or not reasonably available, or when only 1 parent/legal guardian had legal responsibility for the care of the child).

### Infant and mother recruitment

Between November 2023 and August 2025, we recruited infant/mother dyads from urban Los Angeles through digital ads, community events, and provider referrals. Materials were available in English and Spanish. Infants born at term became eligible at 6 mo of age. We excluded infants with endocrine disorders and those with major maternal illness, cognitive or physical limitations, metabolic-related medication use, preterm birth, low birth weight, fetal abnormalities, or maternal age <18 at delivery. Pregnancy complications and infant antibiotic use were not exclusionary.

Feeding strategy was classified using a 24-h maternal dietary recall and formula labels. Infants were classified into 4 feeding strategies: exclusive human milk, exclusive lactose-based formula, exclusive corn syrup–based formula, and mixed (human milk and lactose-based formula). Per IRB protocol, staff contacted each mother ≤3 times before discontinuing outreach. We enrolled a total of 52 infants.

### Data collection

#### Anthropometric and dietary characteristics

Trained clinical research coordinators (CRCs) measured each infant’s weight once using a calibrated scale (Tanita Ironman InnerScan BC-549 Plus) before CGM placement. Mothers provided infant medical records and self-reported clinical information between 3 and 6 mo postpartum, including prepregnancy height and weight, gestational diabetes status, and family history of diabetes in the infant’s parents and grandparents.

Research staff collected up to three 24-h dietary recalls, 2 weekdays and 1 weekend day, by phone during the CGM wear period. Recalls followed the Nutrition Data System for Research (NDSR) multiple-pass method: quick food list, prompts for commonly forgotten items, timing and context of each eating occasion, and detailed descriptions of food and portions. Staff supervised by registered dietitians entered all data into NDSR [24].

#### Continuous glucose monitoring

CRCs applied the FreeStyle Libre Pro (Abbott) CGM to each infant’s upper arm or anterior thigh following IRB-approved procedures and manufacturer instructions. All placements occurred with a parent present, and devices remained in place for 1 wk, extended ≤2 wk if replacement or recollection was needed. CGMs were removed by CRCs or mothers and returned to the study team in person or via courier. CRCs then scanned the device and downloaded raw data from Abbott’s web-based portal (LibreView). The Freestyle Libre Pro has not been validated specifically in infants. Prior studies in pediatric populations have demonstrated reduced CGM accuracy in the hypoglycemic range (≤70 mg/dL), particularly at lower glucose concentrations, compared with euglycemic ranges; thus, hypoglycemic incidents should be interpreted with caution [25,26]. A threshold of ≥140 mg/dL was used to define the hyperglycemic threshold, consistent with prior pediatric CGM literature that uses this cutoff to characterize elevated glucose exposure below overt hyperglycemia [27,28].

#### Data quality control

We processed, analyzed, and visualized all CGM data in R (version 4.5.1; R Core Team, Vienna, Austria) using the packages: tidyverse [29], lubridate [30], e1071 [31], NbClust [32], iglu [33], vegan [34], and fpc [35] (R Core Team, 2025). Each infant’s CGM time series was manually inspected to identify data recording gaps, defined as missing glucose values for >3 consecutive days despite continuous device wear. This pattern, observed in nearly half of participants, consisted of several hours of recorded data immediately after device placement, followed by a multiday recording gap, after which CGM measurements resumed. For affected participants, all glucose values recorded before the gap were excluded from analysis, and only data recorded after the gap were retained. No interpolation or imputation was performed.

### Standardization of 24-h cycles

To avoid bias from partial days (attachments and remove date), we restricted analyses to complete 24-h cycles, ensuring balanced representation of daytime and nighttime glucose values across the cohort. Abbott’s FreeStyle Libre Pro records glucose every 15 min, producing 96 readings per day.

#### Chronology check

We required ≥72 consecutive hours of uninterrupted CGM data per infant to ensure adequate within-subject replication, providing triplicate biological measurements for each of the 96 timepoints per infant [36,37]. Seven infants did not meet this criterion and were excluded, leaving 45 infants for analysis. Data completeness was verified for all participants, and no missing measurements were identified; therefore, imputation of missing data was not required.

#### Data recording gaps

For nearly half of all infants, multiday gaps in CGM recording occurred at the beginning of wear. These gaps were defined as periods when the infant wore the CGM but no glucose values were saved for multiple consecutive days for reasons unknown. After these recording gaps, CGM measurements resume spontaneously. For affected participants, all glucose values recorded before the gap were excluded, and only values recorded after data resumed were retained. No other gaps occurred at any timepoint later during CGM wear.

Values <60 mg/dL were winsorized to align with typical physiological glucose distributions in infants and young children. Clinical reference ranges indicate that children <2 y generally maintain blood glucose values ≥60 mg/dL [38]. CGM profiles in healthy children aged 1 to 6 y show that sensor glucose values <60 mg/dL are uncommon over multiple days, supporting the interpretation that persistent readings below this threshold likely reflect artifact rather than true physiology [39]. These artifacts likely result from “compression lows,” where body pressure on the sensor artificially deflates readings while lying down [40].

To examine whether compression lows occurred more frequently during nighttime compared with daytime, we compared, among infants with compression lows, the number of winsorized low values observed during daytime (07:00–21:59) and nighttime (22:00–06:59) periods using a Wilcoxon signed-rank test. This analysis assessed whether the median number of compression lows differed between periods. Although motion and sleep were not directly measured, compression lows were plausibly more common at night, when infants are more likely to remain recumbent.

### Analytical workflow

#### Computing CGM-derived metrics

We characterized infant glycemic patterns by calculating 28 clinically and research-relevant CGM metrics using iglu [41,42]. These metrics were: estimated hemoglobin A1c (HbA1c); glucose variability; time spent euglycemic (71–139 mg/dL), hypoglycemic (≤70 mg/dL), and hyperglycemic (≥140 mg/dL) [43,44]; area under curve; mean blood glucose; average daily risk range; mean rate of change; continuous glucose monitoring index; continuous overall net glycemic action 1 h and 2 h; glucose management indicator; glycemic risk assessment diabetes equation (GRADE) standard, euglycemic, hypoglycemic, and hyperglycemic; high and low blood glucose indices; hyperglycemic and hypoglycemic indices; index of glycemic control; j index; m value; mean absolute glucose; mean amplitude of glycemic excursions (MAGE); mean of daily differences; and median absolute deviation.

#### Infant feeding strategy analytical framework

Given the observational and exploratory nature of this study, no CGM outcomes were designated as primary endpoints before data collection. Instead, we adopted a hierarchical analytical framework to control multiplicity: *1*) a global permutation multivariate analysis of covariance (MANCOVA) established whether feeding strategy was associated with any multivariate difference in glycemic profiles before metric-level testing was undertaken; *2*) metric-level permutation analysis of covariance (ANCOVAs) were subsequently conducted on the collinearity-reduced subset of CGM metrics, with Benjamini–Hochberg False Discovery Rate (BH-FDR) correction applied across this family of tests; and *3*) dietary and anthropometric comparisons were treated as a separate hypothesis family with independent FDR correction. Clustering and principal component analysis (PCA)-based analyses were explicitly designated as exploratory and interpreted descriptively without formal inference. Freedman–Lane permutation ANCOVA was selected for metric-level inference because it accommodates non-normal outcome distributions, unequal group sizes, and models containing both continuous and categorical covariates. Kruskal–Wallis tests were used for dietary variable comparisons given their non-normal distributions. Ward’s D2 agglomerative hierarchical clustering was selected for its robustness with Euclidean distance metrics and small sample sizes.

To determine whether maternal and infant characteristics differed across feeding strategies in ways that could plausibly influence glycemic regulation, we first compared baseline anthropometric and clinical characteristics across observed feeding strategies (exclusively human milk, lactose-based formula, corn syrup–based formula, or mixed human milk and lactose-based formula). These analyses were descriptive and intended to identify potential sources of confounding rather than to test independent effects on CGM-derived glycemic outcomes. Maternal and infant anthropometric variables included continuous measures (maternal prepregnancy BMI, maternal age, infant age, infant weight, and CGM wear duration) and categorical measures (maternal gestational diabetes status, family history of diabetes, and mode of delivery). Continuous variables were compared across feeding strategies using Kruskal–Wallis tests, and categorical variables were compared using extended Fisher’s exact tests. We included maternal prepregnancy BMI, gestational diabetes status, and family history of diabetes as covariates in subsequent models based on previously demonstrated clinical relevance to glycemic regulation and later risk of type 2 diabetes [45–47].

In subsequent analyses, metric reduction followed a 3-step procedure. First, metrics that reached statistical significance in the overall permutation ANCOVA were identified. Second, pairwise Pearson correlations were computed among those significant metrics across the full sample, without reference to pairwise feeding group differences, to avoid selection bias; metrics exhibiting strong collinearity (*r* > 0.8 or *r* < − 0.8) were grouped into correlate clusters. Third, a single representative metric was selected from each correlate cluster based on 2 criteria applied jointly: *1*) established use in CGM research and clinical literature, and *2*) coverage of a distinct glycemic dimension. Specifically, time spent >140 mg/dL was retained to capture duration of sustained glucose elevation; MAGE was retained to capture the magnitude of within-day glucose excursions; J index was retained as a composite penalty combining mean glucose concentration and variability; and GRADE was retained to capture risk-weighted glucose contribution across the full distribution. Together, these 4 metrics represent complementary and nonredundant glycemic dimensions. Results for all 28 CGM metrics are reported in Supplemental Table 1; the pattern of significant findings was consistent between the full and reduced metric sets. Before metric-level inference, a global permutation-based MANCOVA (9999 permutations) was conducted to evaluate whether the feeding strategy was associated with multivariate differences across the retained CGM-derived glycemic metrics, after covariate adjustment for maternal prepregnancy BMI, gestational diabetes status, family history of diabetes, and mode of delivery. Feeding strategy explained a significant proportion of multivariate variance in glycemic profiles (Pillai’s trace = 0.894, permutation *F* = 2.74, *R*^2^ = 0.164, *P* = 0.004), supporting progression to metric-level permutation ANCOVAs.

To determine whether feeding strategy was associated with differences in CGM-derived measures of glycemic regulation after accounting for biologically relevant maternal risk factors, we compared CGM-derived outcomes across feeding strategies using Freedman–Lane permutational ANCOVA. This approach accommodates unequal group sizes, non-normally distributed outcomes, and models including both categorical and continuous covariates [48]. Given the observational study design, these analyses assessed associations rather than causal effects.

When overall group effects were statistically significant, we conducted permutation-based pairwise comparisons within the same resampling framework, with *P* values adjusted for multiple comparisons using the BH-FDR procedure. To quantify the magnitude of observed differences, effect sizes were reported for all inferential analyses. For the global permutation MANCOVA, we reported Pillai’s trace and *R*^2^ as multivariate effect size indices. For metric-level permutation ANCOVAs, we calculated partial eta squared (_η_^2^_p_) for overall group effects, interpreted using conventional thresholds (_η_^2^_p_ ≈ 0.01, small; 0.06, medium; 0.14, large) [49]. For permutation-based pairwise comparisons, we reported _η_^2^_p_ with 95% bootstrap confidence intervals (BCIs), derived from the same permutation resampling framework, to convey both the magnitude and precision of pairwise group differences. For dietary variable comparisons, we reported effect sizes using eta squared (_η_^2^) for Kruskal–Wallis tests. Uncertainty intervals for all key estimates were obtained via bootstrap resampling (*B* = 9999) to ensure compatibility with the nonparametric and permutation-based analytical framework used throughout.

Post hoc sensitivity analysis indicated that with our sample size (45 infants), ⍺ = 0.05, and accounting for 4 covariates, we had 80% power to detect effects of _η_^2^_p ≥_ 0.203 across our categorical predictors.

We processed and analyzed dietary variables in parallel to further characterize differences across feeding strategies that might relate to glycemic outcomes. For infants with a single day of dietary recall, we used reported nutrient values; for those with 2 or 3 d, we calculated the mean across days. From the NDSR output, we curated infant-relevant dietary variables, including: proportion of solid food intake entries (per day), total energy (kcal); total fat (g); dietary fiber (g); total sugars (g); fructose (g); galactose (g); glucose (g); lactose (g); maltose (g); sucrose (g); starch (g); percentage of calories from fat, carbohydrates, and proteins; % fatty acids (SFA, MUFA, and PUFA); glycemic index (glucose and bread reference); and added sugars by available carbohydrate. Dietary variables were compared across feeding categories using Kruskal–Wallis tests with permutation-based pairwise comparisons and BH-FDR adjustment. To quantify the magnitude of pairwise dietary differences, we reported _η_^2^_p_ with 95% BCIs derived from the same permutation resampling framework. We also examined the consumption of sugar-sweetened beverages and juices across the cohort; only 3 infants were reported to have consumed any such beverages, and because of this very small sample size, these variables were excluded from further analysis.

Given the relatively small sample size across feeding strategies (*n* = 18, 10, 6, and 11), we conducted a post hoc assessment of detectable effect sizes. On the basis of the permutation-based pairwise comparisons, the study had 80% power to detect only large effects (_η_^2^_p ≥_ 0.14). Moderate and small effects may not reach statistical significance at this sample size. Therefore, the absence of statistically significant differences for some outcomes should not be interpreted as evidence of equivalence. Conversely, statistically significant findings should be interpreted in light of the detectable effect size threshold—observed effects that approach this threshold may reflect real differences, but their biological or clinical magnitude warrants cautious interpretation.

#### Reducing glycemic dimensionality and glycemic clusters

To address redundancy among CGM-derived measures, we first computed Pearson correlation coefficients across all 28 CGM-derived metrics. Metrics exhibiting strong pairwise correlations (*r* > 0.8) were considered highly collinear; therefore, we retained a subset of metrics with pairwise correlations constrained to −0.8 < *r* < 0.8 for downstream analyses. This reduced feature set was subsequently scaled and centered.

PCA was then applied as a preprocessing step to reduce dimensionality among the selected subset of noncolinear metrics. Principal components explaining 80% of cumulative variance were retained [50, 51]. The retained principal components were used as inputs for hierarchical clustering to identify data-driven phenotypic groupings of CGM profiles, rather than biologically or mechanistically defined subtypes.

Phenotypic subgroup identification was performed using agglomerative hierarchical clustering with complete linkage and Euclidean distance. The number of clusters was determined by jointly considering the dendrogram, the elbow plot of within-cluster sum of squares, and the mean silhouette width, with all 3 approaches supporting the selection of 4 clusters. Cluster robustness was assessed using bootstrap resampling (*B* = 500), and stability was quantified with the Jaccard similarity coefficient, with all clusters exhibiting Jaccard similarity coefficient values >0.8, suggesting relatively stable groupings within this sample [52,53]. These stability estimates should be interpreted cautiously given the small sample size.

#### Subsequent visual comparisons based on glycemic clusters

In a parallel analysis, we examined CGM-derived metrics, anthropometric variables, and dietary data stratified by glycemic clusters. Rather than performing formal statistical tests, which are underpowered given the sample size, we assessed the distributions of values to identify visual trends across clusters. This approach is descriptive and intended to provide an exploratory overview of potential patterns rather than inferential conclusions.

## Results

### Infant sample characteristics and analytical rationale

During data quality control, winsorizing glucose values did not significantly alter the overall data distribution (Wilcoxon rank-sum; *P* > 0.05; Supplemental Figure 1). Compression lows occurred in 22 infants and were disproportionately higher during nighttime (Wilcoxon signed-rank test; *P* < 0.05; Supplemental Figure 2). Among the 45 infants who passed CGM data quality control, CGM wear duration ranged from 3 to 8 d (mean ± SD: 6.0 ± 1.2 d) (Supplemental Figure 3). Our analytic cohort was ~45% female and 53% male, with a mean age of 28.0 ± 1.3 wk and a mean weight of 8.4 ± 1.2 kg during CGM wear. The descriptive statistics of the sample and subgroups are shown in Table 1.

Feeding categories differed in size, with higher proportions of exclusively human milk (40.0%), lactose formula (22.2%), and CSS formula (24.4%) fed infants, and fewer mixed-fed (human milk and lactose formula) infants (13.3%) (Supplemental Table 2). Feeding strategies did not differ significantly in dyad age and weight or prepregnancy BMI; however, exclusively human milk or mixed-fed infants included mothers with gestational diabetes, and gestational diabetes and blood glucose differed significantly across feeding strategies (Table 1). Mean glucose values appeared descriptively higher in CSS-fed infants compared with other feeding strategies (Table 1).

### CSS-fed infants show higher CGM-derived glycemic variability metrics

After adjusting for prepregnancy BMI, gestational diabetes, family history of diabetes, and proportion of daily solid food intake, 46.4% of CGM-derived metrics differed significantly across feeding strategies [global permutation MANCOVA: Pillai’s trace = 0.894, permutation *F* = 2.74, *R*^2^ = 0.164 (large effect), *P* = 0.004] (Supplemental Table 1). From the 28 available CGM metrics, we selected 4 clinically relevant variables to represent their respective correlative clusters (Supplemental Figure 4): time spent hyperglycemic (>140 mg/dL), GRADE, J index, and MAGE. These metrics were selected for their clinical relevance.

**FIGURE 1.**
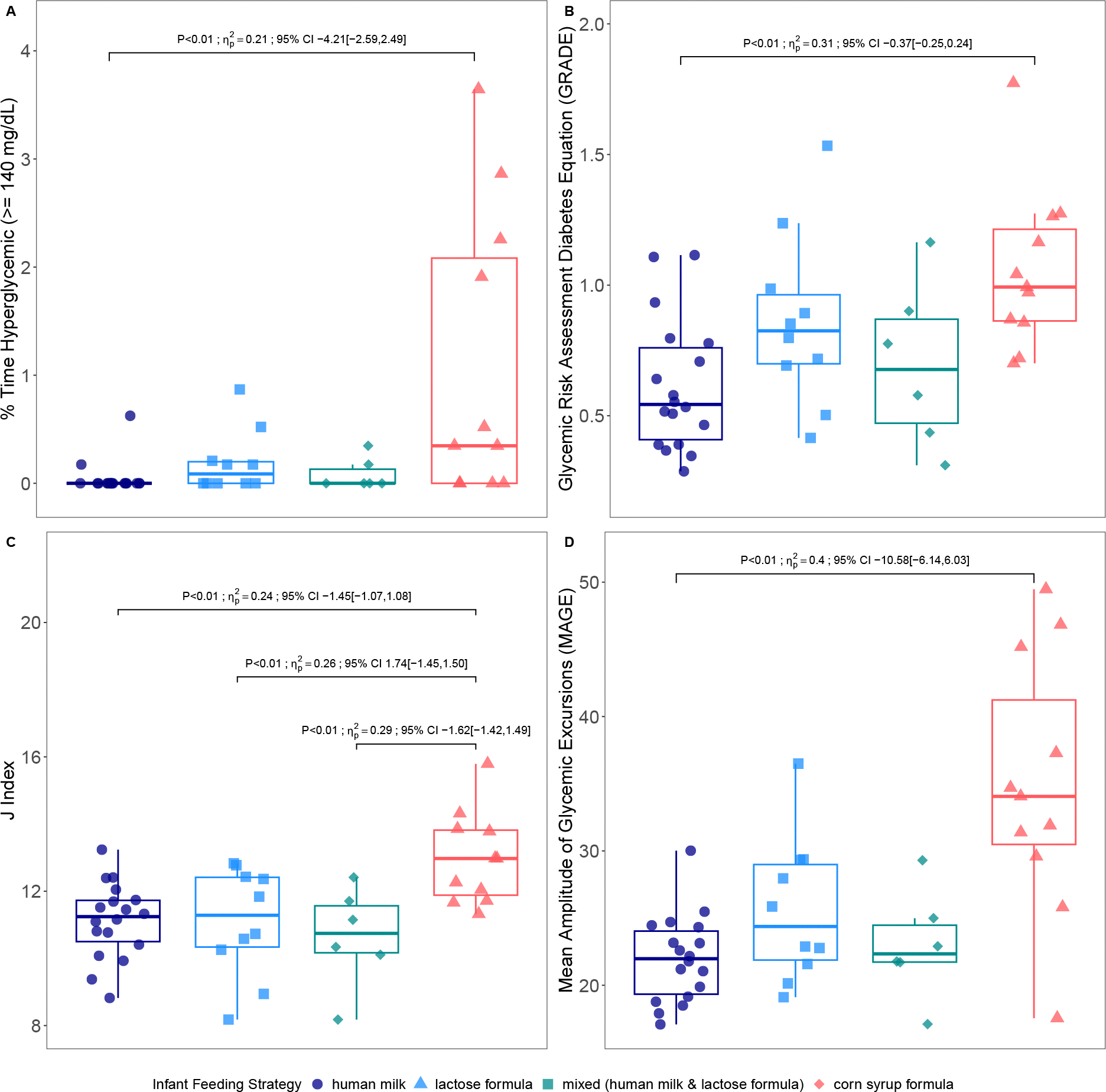
Infants fed formula containing corn syrup solids exhibit consistently and significantly different CGM-derived metrics compared with infants fed human milk. Boxplots display 4 selected CGM-derived metrics across infant feeding strategies. Differences were assessed using permutation-based Freedman–Lane ANCOVA models, covarying for prepregnancy BMI, maternal gestational diabetes, family history of diabetes, and mean daily proportion of solid food entries. Brackets indicate permutation-based post hoc pairwise comparisons displaying FDR-adjusted *P* values (Benjamini–Hochberg method), η^2^_p_ effect sizes, and δ (covariate-adjusted mean difference) with permutation-based 95% bootstrap confidence intervals in brackets. Individual infants are represented as points, color- and shape-coded by feeding strategy. See Supplemental Table 1 for all CGM metric comparisons. ANCOVA, analysis of covariance; CGM, continuous glucose monitors; FDR, false discovery rate.

Infants fed formula containing CSS exhibited significantly higher values and effect sizes for all 4 metrics (time hyperglycemic, GRADE, J index, and MAGE) compared with those fed human milk (Figure 1; _η_^2^_p_ and 95% BCIs for each comparison reported in Figure 1 and Supplemental Table 1). Human milk and CSS formula differed consistently across metrics, whereas other feeding strategies showed significant differences from CSS formula only for J index (Figure 1 and Supplemental Table 1). CSS-based formula-fed infants also demonstrated significant differences from human milk–fed infants in ≥6 additional CGM metrics per comparison (Supplemental Table 1). In contrast, no significant differences emerged among human milk, lactose formula, and mixed feeding strategies across any metrics (_η_^2^_p_ values and 95% BCIs for all significant comparisons reported in Supplemental Table 1).

We next examined whether maternal anthropometric and dietary variables differed across infant feeding strategies. Maternal anthropometric measures, whether continuous or categorical, did not vary significantly by feeding strategy (Supplemental Table 3). In contrast, 85% of infant feeding-relevant dietary variables differed across ≥2 feeding strategies (Table 2). Infants fed CSS formula compared with human milk showed the most consistent contrasts in the expected direction: CSS-fed infants consumed a higher percentage of calories from carbohydrates [*P <* 0.001, _η_^2^_p_ = 0.4, 95% BCI (− 3.72, 3.71)], whereas human milk–fed infants had higher lactose intake [*P <* 0.001, _η_^2^_p_ = 0.57, 95% BCI (− 25.40, 24.90)] (Supplemental Table 4).

### Exploratory hierarchical clustering identifies 4 descriptive glycemic subgroups

To characterize exploratory data-driven glycemic patterns in our infant cohort, we performed PCA on 4 representative CGM-derived metrics from highly correlated metric clusters: time spent ≥140 mg/dL, MAGE, GRADE, and J index. The first 2 principal components explained over 80% of cumulative variance (Supplemental Figure 5) and were used for subsequent cluster analyses. Wards D2 method, silhouette analysis, and bootstrapping converged to identify 4 optimal glycemic clusters (Supplemental Figure 6).

**FIGURE 2.**
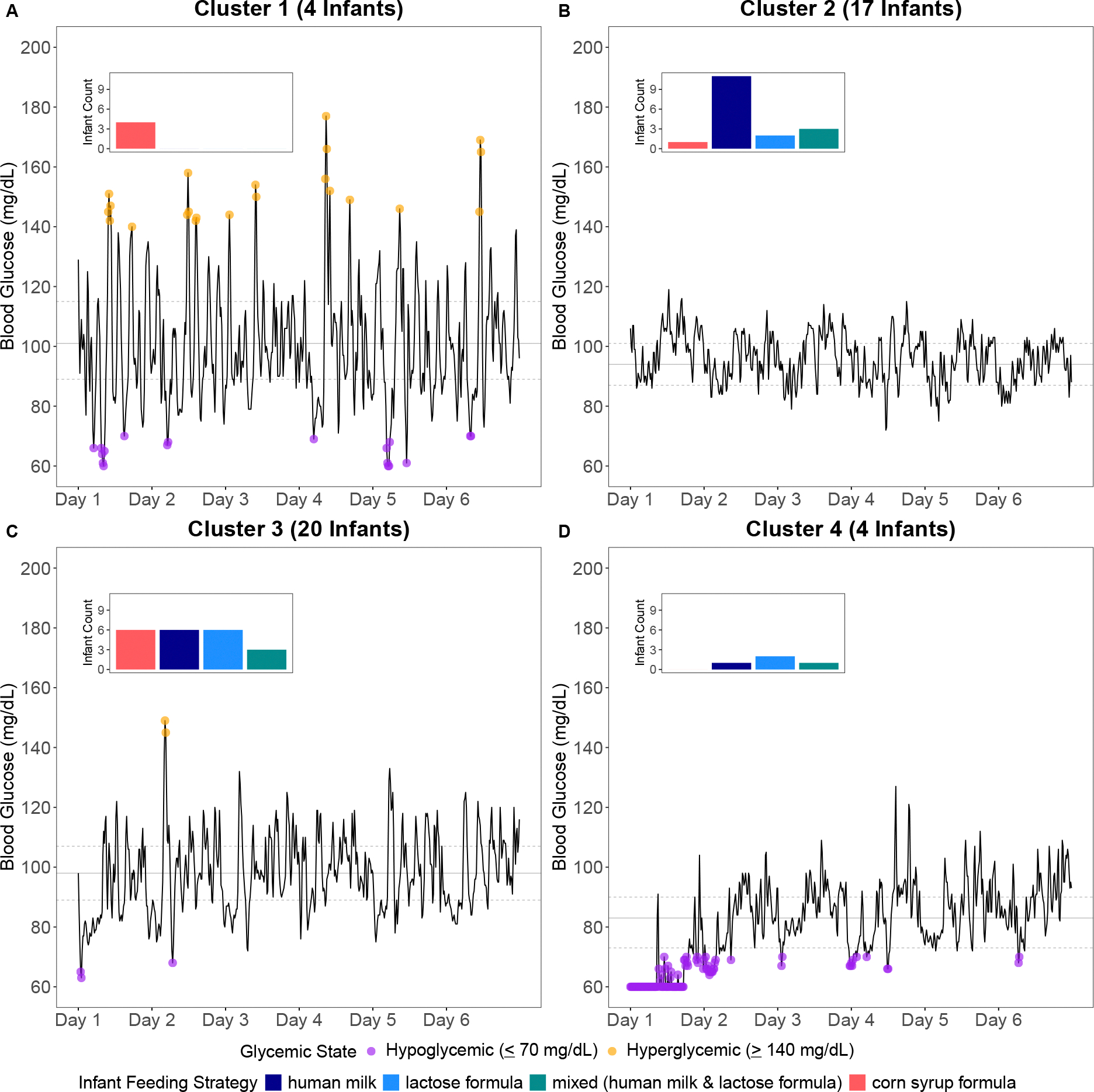
Data-driven exploratory hierarchical clustering reveals 4 infant subgroups with distinct glycemic profiles. Representative individual CGM traces are shown for each cluster subgroup to illustrate nuanced differences in glycemic patterning. Each line plot depicts time-series CGM readings over a 6-d period, with color-coded points highlighting glucose readings >140 mg/dL and <70 mg/dL. Subgroup means and SDs are overlaid as solid and dashed gray lines, respectively. Subgroups are characterized visually and qualitatively as higher glycemic variability (A), lower glycemic variability (B), moderate glycemic variability (C), and more frequent glucose readings <70 mg/dL (D). Bar plots show the distribution of infant feeding strategies within each cluster subgroup. CGM, continuous glucose monitors; PC, principal component.

**FIGURE 3.**
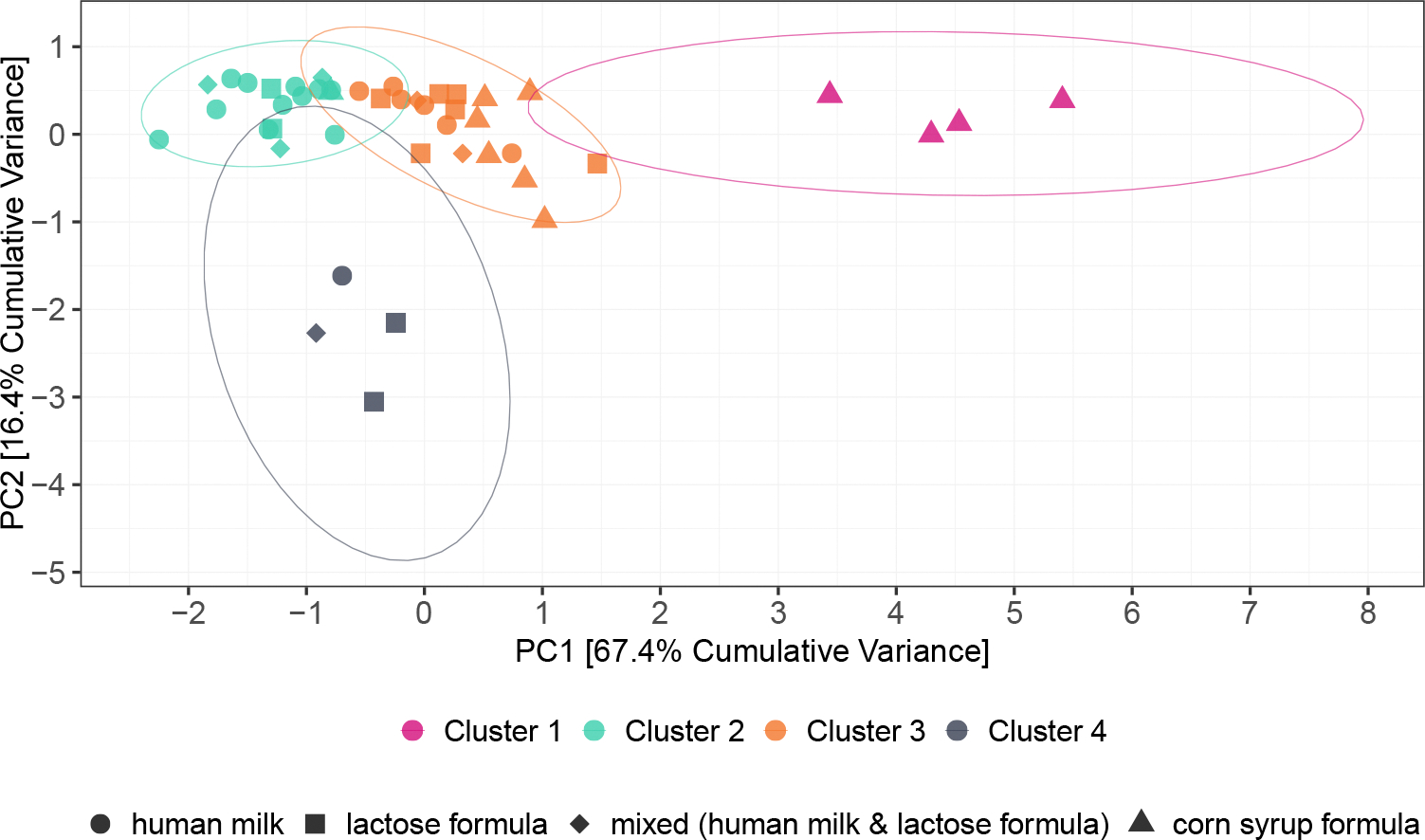
Data-driven exploratory hierarchical clusters exhibit distinct compositional differences in CGM-derived metrics. PCA of 4 scaled and centered CGM-derived metrics (MAGE, GRADE, J index, and percentage time >140 mg/dL) across all infants (n = 45). Points are color-coded by hierarchical cluster and shape-coded by feeding strategy. Ellipses represent 95% concentration ellipses per cluster, shown for descriptive visualization of distributional spread and should not be interpreted as inferential confidence intervals. CGM, continuous glucose monitors; GRADE, glycemic risk assessment diabetes equation; MAGE, mean amplitude of glycemic excursions; PCA, principal component analysis.

The 4 clusters exhibited descriptively distinct patterns: higher glycemic variability metrics (cluster 1; *n* = 4), lower glycemic variability metrics (cluster 2; *n* = 17), moderate glycemic variability metrics (cluster 3; *n* = 20), and more frequent glucose readings <70 mg/dL (cluster 4; *n* = 4) (Figure 2 and Supplemental Figure 7). Cluster 1 consisted exclusively of CSS formula–fed infants, whereas clusters 2 and 3 included infants from all feeding strategies. (Figure 2 and Supplemental Figure 7). In the PCA biplot, cluster 1 appeared more separated from the other clusters in multivariate space; this visual pattern is descriptive and should not be interpreted as a statistical validation of cluster distinctness (Figure 3).

**FIGURE 4.**
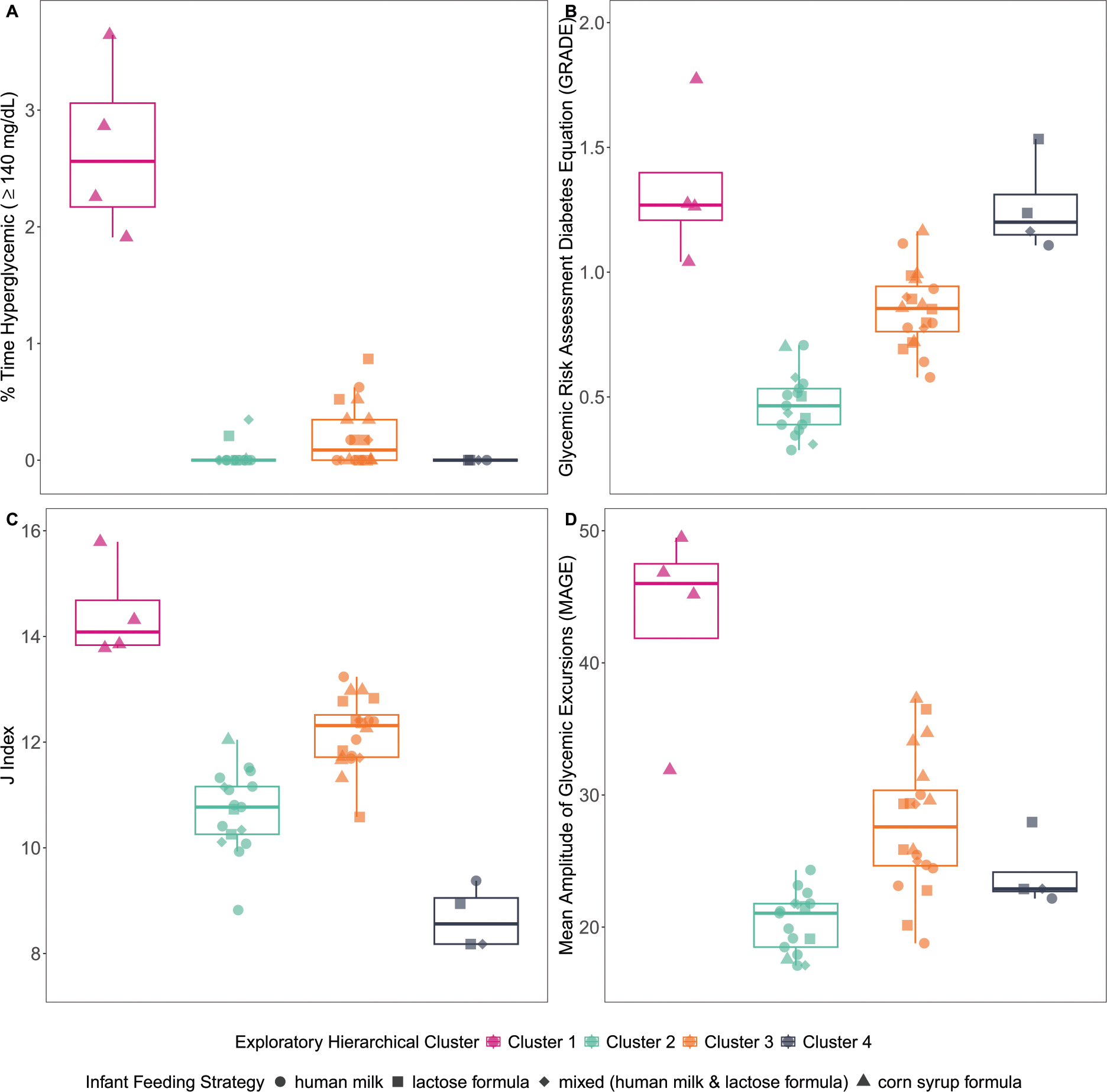
The identified higher glycemic variability cluster exhibits descriptively distinct CGM-derived glycemic metric distributions compared with all other clusters. Boxplots display 4 selected CGM-derived metrics across data-driven hierarchical clusters, presented for visual inspection of distributional differences. Points are color-coded by cluster and shape-coded by feeding strategy. See Supplemental Table 4 for all CGM metric comparisons. CGM, continuous glucose monitors.

Mirroring the visualization conducted for infant feeding strategies, the distribution of time spent *>*140 mg/dL, MAGE, GRADE, and J index showed that cluster 1 had descriptively higher values than all other clusters, particularly clusters 2 and 3 (Figure 4 and Supplemental Table 5). Although we did not conduct pairwise comparisons between clusters due to insufficient power for hierarchical clustering analyses, visual inspection of medians and IQRs showed that infants in the high-variability cluster had higher HbA1c (Supplemental Table 5). Similarly, dietary inspection revealed descriptive differences between cluster 1 and other clusters, despite the absence of formal statistical testing. Cluster 1 infants consumed a higher proportion of solid foods and exhibited elevated intake of fructose, glucose, sucrose, and starch compared with infants in clusters 2, 3, and 4 (Supplemental Table 6).

## Discussion

This pilot study represents the first use of CGMs to explore glucose responses in infants exposed to different feeding strategies. By capturing several full days of CGM data, we provide preliminary insight into infant glycemic regulation during a critical developmental period. Our exploratory analyses suggest that infants fed formula containing CSS exhibit more glycemic variability than traditional formula–fed or human milk–fed infants. This pattern highlights the potential role of carbohydrate type in infant formula, specifically, the inclusion of CSS in shaping glucose regulation, though larger studies are needed to confirm these observations. These preliminary results align with emerging evidence that early exposure to nonlactose carbohydrates may be associated with differences in infant metabolic function [14,17,18]. However, given the modest sample size and recruitment from a specific population, the generalizability of these findings may be limited. Replication in larger and more socioeconomically, racially, and geographically diverse cohorts will be necessary to determine whether these glycemic patterns are consistent across broader infant populations. Accordingly, these findings should be interpreted as preliminary and hypothesis generating, and should not be used to inform clinical recommendations regarding formula selection.

In this context, it is also important to consider the study’s statistical power when interpreting both significant and nonsignificant results. The sample size provided 80% power to detect only large effects (_η_^2^_p_ ≥ 0.203), indicating that moderate or small differences between feeding groups may have gone undetected. As such, nonsignificant comparisons should not be interpreted as evidence of equivalence across outcomes. Although statistically significant findings exceeded our detection threshold, they do not, on their own, establish biological importance or clinical relevance.

Data-driven hierarchical clustering revealed 4 exploratory glycemic clusters that were not fully explained by feeding strategy alone. Nearly half of the CSS-fed infants displayed greater CGM-derived glycemic variability, marked by frequent excursions, elevated variability, repeated episodes of elevated glucose (≥40 mg/dL increase within 1 h), and subsequent transient rapid declines in blood glucose [54]. Although the small sample size limits definitive conclusions, these exploratory findings suggest individual heterogeneity in response to glucose polymers, potentially reflecting differences in absorption kinetics or immature β-cell regulatory capacity. Future studies at larger scales are needed to identify the physiological and dietary factors that contribute to this variability, examine how these patterns evolve with growth, and determine whether they predict future metabolic risk, including type 2 diabetes.

Notably, we observed no significant differences in glycemic metrics among infants fed human milk, lactose-based formula, or mixed feeding strategies, indicating that differences are driven primarily by CSS consumption. Most maternal anthropometrics did not differ across feeding strategies, suggesting that most factors did not confound our findings. Although we did not conduct a formal dietary pattern analysis, review of dietary recall entries indicated that most solid food entries consisted of baby foods or vegetables, and no infants consumed sugar-sweetened beverages. This contextual detail is important given that the median and interquartile range of CSS-fed infants were nearly double those of human milk–fed infants despite the absence of statistically significant results. These null results underscore the specificity of the effects associated with CSS exposure, rather than general formula feeding.

Although previous work shows that lactose-reduced formula supports normal growth [55,56], our study expands the literature by addressing metabolic outcomes that have received less attention. Prior research from our group has linked corn syrup-containing formulas to increased food fussiness [57], altered gut microbiota [15], and higher intake of sugar-sweetened beverages [58]. We also reported a 10% higher obesity risk among infants in California’s Women, Infants & Children Program who were fed corn syrup formulas [13]. Although each study has limitations, the convergence of these findings across multiple outcomes suggests that early exposure to glucose polymers warrants further investigation for its potential impact on metabolic pathways and long-term dietary behaviors.

Because this study is observational and exploratory, unmeasured confounders, such as parental feeding decisions or subtle differences in formula composition, may impact our results. The small sample size and absence of randomized controlled trials limit causal interference, although ethical constraints make such trials difficult in infant populations. Nevertheless, the accumulating observational evidence on the metabolic effects of corn syrup–based formulas suggests a need to more rigorously examine infant feeding guidelines related to formula composition. Formulas made with CSSs contain carbohydrate sources not found in human milk, and when present in any other food would be classified as added sugars under current U.S. Food and Drug Administration / U.S. Department of Agriculture definitions, and Dietary Guidelines for Americans advise against added sugars during infancy and early childhood. As infant feeding practices evolve, evaluating the metabolic implications of formula composition remains essential.

This study has several limitations. The modest sample size warrants caution in interpreting findings, and the lack of long-term follow-up limits our ability to assess persistent metabolic effects of early feeding practices. Three additional limitations warrant explicit acknowledgment. First, the feeding group assignment was not randomized; families self-selected into feeding strategies, meaning unmeasured differences in parental behavior, socioeconomic factors, or infant characteristics may confound observed associations, and residual confounding cannot be excluded even after covariate adjustment. Second, the number of outcomes and analyses conducted across 28 CGM metrics, multiple feeding group comparisons, and exploratory clustering increases the probability of chance findings; although a hierarchical analytical framework with FDR correction was applied to limit this risk, some significant results may reflect type I error. Third, the cross-sectional design characterizes glucose patterns at a single developmental window and cannot speak to whether these patterns persist, resolve, or predict later metabolic outcomes.

Several CGM metrics, including MAGE, J Index, GRADE, and time in hyperglycemic range (≥140 mg/dL, aligned with values reported in nondiabetic pediatric populations [39,59,60], suggesting the observed glycemic patterns do not indicate overt clinical risk by established reference standards. However, these reference data derive primarily from older children and adolescents rather than infants, limiting direct comparability. Whether early glycemic patterns predict later metabolic outcomes remains unknown, as longitudinal data are unavailable in this study and represent a critical gap for downstream research. Future research should prioritize replication in larger, diverse cohorts with long-term outcomes such as insulin sensitivity, adiposity, and obesity risk. Mechanistic studies exploring gut–microbiome composition, insulin dynamics, and macronutrient interactions could clarify how carbohydrate sources influence glucose regulation and infant metabolism.

Overall, our findings demonstrate statistical differences in CGM-derived glycemic variability metrics between human milk–fed infants and those fed CSS-containing formulas. CSS-fed infants exhibited greater glycemic variability and more variable glucose patterns, including more frequent glucose excursions >140 mg/dL and <70 mg/dL. Using an unsupervised, data-driven approach, we identified distinct glycemic subgroups, with the highest-variability cluster composed exclusively of CSS-fed infants, highlighting a subset with markedly higher glycemic variability metrics. These results underscore the heterogeneity of glucose responses in infancy and raise important questions about the long-term metabolic implications of early formula composition, particularly regarding obesity and type 2 diabetes risk. Given the continued prevalence of formula feeding, further research is critical to guide evidence-based recommendations that support optimal metabolic and developmental health in infants.

## Supplementary Material

MMC1

## Figures and Tables

**TABLE 1:** Anthropometric factors of the infant/mother dyad study population

Anthropometric factors	Total (*N* = 45)	Human milk (*n* = 18)	Lactose formula (*n* = 10)	Mixed (human milk and lactose formula) (*n* = 6)	Corn syrup formula (*n* = 11)	*P* value

Maternal						
Age (y)	29.2 (27.7–33.6)	30.1 (28.3–36.3)	27.7 (23.5–30.8)	33.5 (30.9–38.1)	33.8 (29.2–37.4)	0.17
Prepregnancy BMI	29.3 (26.6–35.1)	29.3 (25.9–32.8)	29.2 (27.4–30.0)	30.3 (28.3–38.4)	35.1 (26.7–38.5)	0.62
Gestational diabetes^1^	7.0 (15.6%)	4.0 (22.2%)	0.0 (0.0%)	3.0 (50.0%)	0.0 (0.0%)	<0.05
Infant						
Age (wk)	27.9 (26.9–29.1)	27.9 (26.6–29)	28.5 (27.8–29)	28.5 (28–29.1)	27.0 (26.6–28.9)	0.64
Weight (kg)	8.3 (7.5–8.9)	8.0 (7.12–8.7)	8.7 (8–9.17)	8.6 (8.3–8.67)	8.1 (7.8–8.95)	0.63
Blood glucose (mg/dL)	96.0 (92.3–99.7)	96.0 (92.5–98.1)	93.5 (90.0–100.2)	93.7 (90.2–95.4)	100.2 (95.7–101.4)	0.20
Family history of diabetes^1^	26.0 (57.8%)	10.0 (55.6%)	3.0 (30.0%)	5.0 (83.3%)	8.0 (72.7%)	0.15

Maternal and infant anthropometric factors are summarized as median (IQR) for continuous variables, and as *n* (%) for the categorical variables. Kruskal-Wallis assesses significant differences across feeding strategies with FDR-adjusted *P* values (Benjamini-Hochberg).

Effect sizes (η^2^) are not reported for Table 1 variables, as these comparisons are descriptive and intended to characterize the sample rather than test inferential hypotheses.

Abbreviation: FDR, false discovery rate.

1 Categorical variables are binary (yes/no).

**TABLE 2:** Select dietary characteristics of infant feeding strategies based on maternal 24 h recalls

Dietary factors	Total	Human milk	Lactose formula	Mixed (human milk and lactose formula)	Corn syrup formula	Kruskal-Wallis		
	*N* = 45	*n* = 18	*n* = 10	*n* = 6	*n* = 11	*χ* ^2^	*P*	η^2^_p_

Energy (kcal)	716.92 (616.82–866.70)	746.99 (644.12–877.00)	672.16 (567.81–746.54)	780.51 (651.07–873.92)	649.51 (613.71–803.77)	2.57	0.46	0.01
Total fat (g)	38.72 (32.96–49.30)	45.00 (37.98–54.48) ^A^	34.24 (27.18–40.56) ^B^	44.85 (37.67–51.20) ^A,B^	34.31 (29.25–40.13) ^B^	10.25	<0.05	0.20
Total dietary fiber (g)	2.30 (0.68–5.81)	1.01 (0.44–1.87) ^A^	8.14 (5.45–9.12) ^B^	4.33 (3.13–6.29) ^A,B^	2.58 (1.35–5.57) ^A,B^	15.07	<0.01	0.31
Calories from fat (%)	48.56 (44.59–53.29)	53.60 (51.85–55.00) ^A^	45.70 (44.96–47.09) ^B^	50.12 (49.41–52.17) ^A,C^	43.35 (42.73–46.04) ^B,D^	24.00	<0.001	0.52
Calories from carbohydrates (%)	42.20 (39.42–47.34)	39.35 (38.09–41.43) ^A^	43.78 (41.78–47.15) ^B,C^	42.20 (40.53–42.79) ^A,B^	47.86 (45.65–48.55) ^C^	18.77	<0.001	0.40
Calories from protein (%)	7.61 (6.47–8.44)	6.28 (6.24–6.55) ^A^	8.37 (7.93–8.58) ^B^	7.18 (7.00–7.34) ^A^	8.44 (7.86–9.18) ^B^	27.19	<0.001	0.60
Calories from SFAs (%)	20.28 (14.07–23.69)	24.32 (23.46–24.96) ^A^	11.52 (10.66–20.06) ^B^	17.76 (17.43–19.67) ^B^	14.18 (13.28–17.81) ^B^	28.32	<0.001	0.63
Calories from MUFAs (%)	19.96 (17.23–21.71)	20.34 (19.51–20.82) ^A^	22.59 (13.42–24.98) ^A^	21.92 (21.54–22.56) ^A^	17.25 (16.43–17.70) ^B^	13.25	<0.01	0.27
Calories from PUFAs (%)	7.03 (6.20–8.69)	6.19 (5.98–6.24) ^A^	8.71 (8.66–8.93) ^B^	7.28 (6.86–7.56) ^A,C^	8.49 (8.39–8.84) ^B,C^	27.40	<0.001	0.60
Glycemic index (glucose reference)	43.10 (41.36–52.99)	41.74 (41.00–42.88) ^A^	42.42 (39.71–45.64) ^A^	43.10 (41.98–43.32) ^A^	86.37 (84.17–89.63) ^B^	24.93	<0.001	0.55
Glycemic index (bread reference)	61.62 (59.14–75.77)	59.69 (58.63–61.32) ^A^	60.61 (56.78–65.21) ^A^	61.62 (60.02–61.91) ^A^	123.40 (120.26–128.05) ^B^	24.93	<0.001	0.55
Total sugars (g)	67.82 (50.09–81.50)	76.61 (67.32–91.31) ^A^	71.37 (53.67–74.61) ^A^	74.84 (67.28–84.18) ^A^	38.94 (35.54–50.95) ^B^	20.25	<0.001	0.43
Fructose (g)	1.68 (0.14–4.16)	0.52 (0.01–3.47)	1.10 (0.08–5.28)	1.91 (0.53–2.50)	3.74 (1.73–6.10)	3.45	0.35	0.03
Galactose (g)	0.01 (0.00–0.02)	0.00 (0.00–0.00) ^A^	0.05 (0.03–0.22) ^B^	0.01 (0.01–0.01) ^A,B,C^	0.01 (0.00–0.02) ^A,C^	18.70	<0.001	0.40
Glucose (g)	2.21 (0.31–7.64)	0.36 (0.06–2.51) ^A^	4.10 (0.74–5.67) ^A^	1.47 (0.55–1.94) ^A^	13.10 (11.95–15.99) ^B^	22.25	<0.001	0.48
Lactose (g)	59.32 (37.96–75.46)	70.62 (59.32–84.75) ^A^	57.96 (47.31–71.88) ^A^	69.73 (64.13–80.04) ^A^	0.16 (0.12–6.10) ^B^	25.84	<0.001	0.57
Maltose (g)	0.04 (0.00–1.22)	0.01 (0.00–0.04) ^A^	0.06 (0.00–0.54) ^A^	0.01 (0.00–0.05) ^A^	7.73 (5.92–9.57) ^B^	24.65	<0.001	0.54
Sucrose (g)	1.55 (0.33–5.14)	0.46 (0.01–1.98) ^A^	1.15 (0.14–2.99) ^A^	1.92 (1.00–2.10) ^A^	12.90 (6.38–15.53) ^B^	14.91	<0.01	0.31
Starch (g)	0.90 (0.14–3.53)	0.86 (0.01–2.66)	0.59 (0.01–1.58)	1.93 (0.23–4.52)	2.40 (0.75–10.78)	4.97	0.19	0.07
Added sugars by available carbohydrate (g)	18.15 (0.05–47.18)	0.00 (0.00–0.18) ^A^	35.84 (29.38–51.37) ^B^	18.15 (13.65–23.05) ^B^	60.32 (45.63–74.01) ^C^	34.49	<0.001	0.77
Dietary recall solid food intake (%)	15.94 (8.04–23.96)	12.73 (8.33–18.70)	11.11 (5.91–21.58)	14.93 (9.64–20.01)	26.26 (15.15–35.98)	4.95	0.19	0.05

Factors are expressed as median (IQR) for dietary variables. Superscripted letters (A, B, and C) denote significant differences across pairwise comparisons via permutation-based pairwise post hoc test and Benjamini-Hochberg false discovery rate correction.

Abbreviations: *χ*^2^, chi square; η^2^, effect size.

## Data Availability

Data, code, and analysis, described in the manuscript, code book, and analytic code will be made available on request pending approval by the corresponding author, Michael I. Goran.

## Appendix A. Supplementary data

Supplementary data to this article can be found online at https://doi.org/10.1016/j.ajcnut.2026.101325.

## Abbreviations:

ANCOVA: analysis of covariance
95% BCI: 95% bootstrapped confidence interval
BH-FDR: Benjamini–Hochberg False Discovery Rate
CGM: continuous glucose monitor
CRC: clinical research coordinator
CSS: corn syrup solids
GRADE: glycemic risk assessment diabetes equation
HbA1c: hemoglobin A1c
IRB: Institutional Review Board
MAGE: mean amplitude of glycemic excursions
MANCOVA: multivariate analysis of covariance
NDSR: Nutrition Data System for Research
PCA: principal component analysis

## References

[1] Infant Formula Act of 1980, Pub. L. No. 96–359, 94 Stat. 1190 (1980) (codified as amended at 21 U.S.C. §350a). H.R. 6940, 96th Cong. (1980).

[2] Infant Formula Labeling Requirements, 21 C.F.R pt. 107, Available from: Electronic Code of Federal Regulations (eCFR), 2025.

[3] Imdad A, Sherwani R, Wall K, Pediatric formulas: an update, Pediatr. Rev. 45 (2024) 394–405. 38945989 10.1542/pir.2023-006002

[4] Report No.: CODEX STAN, Codex Standard for Infant Formula and Formulas for Special Medical Purposes Intended for Infants [Internet]. Rome (FAO headquarters), Food and Agriculture Organization of the United Nations / World Health Organization (FAO/WHO), 1981, pp. 72–1981. Available from: https://www.fao.org/fao-who-codexalimentarius/sh-proxy/en/?lnk=1&url=https%253A%252F%252Fworkspace.fao.org%252Fsites%252Fcodex%252FStandards%252FCXS%2B72-1981%252FCXS_072e.pdf.

[5] Strzalkowski AJ, Järvinen KM, Schmidt B, Young BE, Protein and carbohydrate content of infant formula purchased in the United States, Clin. Exp. Allergy 52 (2022) 1291–1301. 36129802 10.1111/cea.14232

[6] Clouard C, Le Bourgot C, Respondek F, Bolhuis JE, Gerrits WJJ, A milk formula containing maltodextrin, vs. lactose, as main carbohydrate source, improves cognitive performance of piglets in a spatial task, Sci. Rep. 8 (2018) 9433. 29930401 10.1038/s41598-018-27796-1 PMC6013478

[7] Wright CJ, Atkinson FS, Ramalingam N, Buyken AE, Brand-Miller JC, Effects of human milk and formula on postprandial glycaemia and insulinaemia, Eur. J. Clin. Nutr. 69 (2015) 939–943. 25804277 10.1038/ejcn.2015.29

[8] Vos MB, Kaar JL, Welsh JA, Van Horn LV, Feig DI, Anderson CAM, , Added sugars and cardiovascular disease risk in children: a scientific statement from the American Heart Association, Circulation 135 (2017) e1017–e1034. Available from: https://www.ahajournals.org/doi/10.1161/CIR.0000000000000439. 27550974 10.1161/CIR.0000000000000439 PMC5365373

[9] DiMaggio DM, Abersone I, Porto AF, Infant consumption of 100% lactose-based and reduced lactose infant formula in the United States: review of NHANES data from 1999 to 2020, J. Pediatr. Gastroenterol. Nutr. 79 (2024) 1017–1023. 38934419 10.1002/jpn3.12292

[10] Lewis JI, Dror DK, Hampel D, Kac G, Mølgaard C, Moore SE, , Reference values for macronutrients in human milk: the mothers, infants and lactation quality (MILQ) study, Adv. Nutr. 16 (2025) 100501. 41167834 10.1016/j.advnut.2025.100501 PMC12673390

[11] Russell MJ, Should we be concerned about the use of lactose-free infant formulas? J. Pediatr. Gastroenterol. Nutr. 79 (2024) 929–933. 39315662 10.1002/jpn3.12375

[12] Slupsky CM, He X, Hernell O, Andersson Y, Rudolph C, Lönnerdal B, , Postprandial metabolic response of breast-fed infants and infants fed lactose-free vs regular infant formula: a randomized controlled trial, Sci. Rep. 7 (2017) 3640. 28623320 10.1038/s41598-017-03975-4 PMC5473881

[13] Anderson CE, Whaley SE, Goran MI, Lactose-reduced infant formula with corn syrup solids and obesity risk among participants in the special supplemental nutrition program for women, infants, and children (WIC), Am. J. Clin. Nutr. 116 (2022) 1002–1009. 35998087 10.1093/ajcn/nqac173 PMC10157812

[14] Mokhtari P, Schmidt KA, Babaei M, Goran MI, Altered nutrient composition of lactose-reduced infant formula, Nutrients 16 (2024) 276. 38257168 10.3390/nu16020276 PMC10821187

[15] Jones RB, Berger PK, Plows JF, Alderete TL, Millstein J, Fogel J, , Lactose-reduced infant formula with added corn syrup solids is associated with a distinct gut microbiota in Hispanic infants, Gut Microbes 12 (2020) 1813534. 32887539 10.1080/19490976.2020.1813534 PMC7524300

[16] Hampson HE, Jones RB, Berger PK, Plows JF, Schmidt KA, Alderete TL, , Adverse effects of infant formula made with corn-syrup solids on the development of eating behaviors in Hispanic children, Nutrients 14 (2022) 1115. 35268090 10.3390/nu14051115 PMC8912730

[17] Anderson CE, Goran MI, Whaley SE, Any infant formula amount, but not infant formula type, is associated with less healthful subsequent beverage intake among special supplemental nutrition program for women, infants, and children–participating children, Curr. Develop. Nutr. 8 (2024) 102094. 10.1016/j.cdnut.2024.102094 PMC10897848 38419833

[18] Rani U, Alwasila R, Story WT, Ten Eyck P, Hoberg A, Santillan DA, , Effects of infant formula type on early childhood growth outcomes: a retrospective cohort study, Nutrients 17 (2025) 3111. 41097188 10.3390/nu17193111 PMC12525715

[19] Galicia-Garcia U, Benito-Vicente A, Jebari S, Larrea-Sebal A, Siddiqi H, Uribe KB, , Pathophysiology of type 2 diabetes mellitus, IJMS 21 (2020) 6275. 32872570 10.3390/ijms21176275 PMC7503727

[20] Hauschild M, Monnard C, Eldridge AL, Antoniou MC, Bouthors T, Hansen E, , Glucose variability in 6–12-month-old healthy infants, Front. Nutr. 10 (2023) 1128389. 37502727 10.3389/fnut.2023.1128389 PMC10369064

[21] Harris DL, Weston PJ, Harding JE, Relationships between feeding and glucose concentrations in healthy term infants during the first five days after birth—the Glucose in Well Babies Study (GLOW), Front. Pediatr. 11 (2023) 1147659. 37033167 10.3389/fped.2023.1147659 PMC10079951

[22] Shah R, McKinlay CJD, Harding JE, Neonatal hypoglycemia: continuous glucose monitoring, Curr. Opin. Pediatr. 30 (2018) 204–208. 29346140 10.1097/MOP.0000000000000592 PMC5882205

[23] McKinlay CJD, Chase JG, Dickson J, Harris DL, Alsweiler JM, Harding JE, Continuous glucose monitoring in neonates: a review, Matern. Health, Neonatol. Perinatol. 3 (2017) 18. 29051825 10.1186/s40748-017-0055-z PMC5644070

[24] University of Minnesota, Nutrition Data System for Research (NDSR). Minneapolis, MN: University of Minnesota.

[25] de Bock M, Codner E, Huynh T, Maahs DM, Mahmud FH, Marcovecchio L, , ISPAD clinical practice consensus guidelines 2022: glycemic targets and glucose monitoring for children, adolescents, and young people with diabetes, Pediatr, Diabetes 23 (2022) 1270–1276. 36537523 10.1111/pedi.13455 PMC10107615

[26] Ghane N, Broadney MM, Davis EK, Trenschel RW, Collins SM, Brady SM, , Estimating plasma glucose with the FreeStyle libre pro continuous glucose monitor during oral glucose tolerance tests in youth without diabetes, Pediatr, Diabetes 20 (2019) 1072–1079. 31433542 10.1111/pedi.12910 PMC6821586

[27] Chobot A, Piona C, Bombaci B, Kaminska-Jackowiak O, Mancioppi V, Passanisi S, Exploring the continuous glucose monitoring in pediatric diabetes: current practices, innovative metrics, and future implications, Children 11 (2024) 907. 39201842 10.3390/children11080907 PMC11352692

[28] Marco A, Pazos-Couselo M, Moreno-Fernandez J, Díez-Fernandez A, Alonso-Sampedro M, Fernandez-Merino C, , Time above range for predicting the development of type 2 diabetes, Front. Public Health 10 (2022) 1005513. 36568777 10.3389/fpubh.2022.1005513 PMC9772988

[29] Wickham H, Averick M, Bryan J, Chang W, McGowan L, François R, , Welcome to the Tidyverse, JOSS 4 (2019) 1686.

[30] Grolemund G, Wickham H, Dates and times made easy with lubridate, J. Stat. Soft. 40 (2011) 1–25. Available from: http://www.jstatsoft.org/v40/i03/.

[31] Meyer D, Dimitriadou E, Hornik K, Weingessel A, Leisch F, E1071: misc functions of the department of statistics, probability theory group (Formerly: E1071), TU Wien, 1999, pp. 1.7–16 [cited 3 July, 2025]. Available from: https://CRAN.R-project.org/package=e1071.

[32] Charrad M, Ghazzali N, Boiteau V, Niknafs A, NbClust: an R package for determining the relevant number of clusters in a data set, J. Stat. Soft. 61 (2014) 1–36. Available from: http://www.jstatsoft.org/v61/i06/.

[33] Broll S, Urbanek J, Buchanan D, Chun E, Muschelli J, Punjabi NM, , Interpreting blood GLUcose data with R package iglu, PLOS ONE 16 (2021) e0248560. 33793578 10.1371/journal.pone.0248560 PMC8016265

[34] Dixon P, VEGAN, a package of R functions for community ecology, J. Vegetation Sci. 14 (2003) 927–930.

[35] Henning C. fpc: Flexible Procedures for Clustering. R package version 2.2–12; 2020. Available from CRAN Package Repository - fpc.

[36] Zhang S, Ahn C, How many measurements for time-averaged differences in repeated measurement studies? Contemp. Clin. Trials. 32 (2011) 412–417. 21241827 10.1016/j.cct.2011.01.002 PMC3070039

[37] Grunwald GK, Sullivan DK, Hise M, Donnelly JE, Jacobsen DJ, Johnson SL, , Number of days, number of subjects, and sources of variation in longitudinal intervention or crossover feeding trials with multiple days of measurement, Br. J. Nutr. 90 (2003) 1087–1095. 14641968 10.1079/bjn2003989

[38] Rosenfeld E, Thornton PS, Hypoglycemia in infants and children, Endotext, MDText.com, Inc, South Dartmouth (MA), 2023.

[39] DuBose SN, Kanapka LG, Bradfield B, Sooy M, Beck RW, Steck AK, Continuous glucose monitoring profiles in healthy, nondiabetic young children, J. Endocr. Soc. 6 (2022) bvac060. 35506147 10.1210/jendso/bvac060 PMC9049110

[40] Facchinetti A, Del Favero S, Sparacino G, Cobelli C, Modeling transient disconnections and compression artifacts of continuous glucose sensors, Diabetes Technol. Ther 18 (2016) 264–272. 26882463 10.1089/dia.2015.0250

[41] Zahalka SJ, Galindo RJ, Shah VN, Low Wang CC, Continuous glucose monitoring for prediabetes: what are the best metrics? J. Diabetes Sci. Technol. 18 (2024) 835–846. 38629784 10.1177/19322968241242487 PMC11307227

[42] Battelino T, Alexander CM, Amiel SA, Arreaza-Rubin G, Beck RW, Bergenstal RM, , Continuous glucose monitoring and metrics for clinical trials: an international consensus statement, Lancet Diabetes Endocrinol 11 (2023) 42–57. 36493795 10.1016/S2213-8587(22)00319-9

[43] Pressig CM, Rigby MR, Pediatric critical illness hyperglycemia: risk factors associated with development and severity of hyperglycemia in critically ill children, J. Pediatr. 155 (2009) 734–739. 19628220 10.1016/j.jpeds.2009.05.007

[44] Umpierrez GE, Hellman R, Korytkowski MT, Kosiborod M, Maynard GA, Montori VM, , Management of hyperglycemia in hospitalized patients in non-critical care setting: an endocrine society clinical practice guideline, J. Clin. Endocrinol. Metab. 97 (2012) 16–38. 22223765 10.1210/jc.2011-2098

[45] Dabelea D, Hanson RL, Lindsay RS, Pettitt DJ, Imperatore G, Gabir MM, , Intrauterine exposure to diabetes conveys risks for type 2 diabetes and obesity: a study of discordant sibships, Diabetes 49 (2000) 2208–2211. 11118027 10.2337/diabetes.49.12.2208

[46] Barker DJP, The developmental origins of adult disease, J. Am. Coll. Nutr. 23 (2004) 588S–595S. 15640511 10.1080/07315724.2004.10719428

[47] Gaillard R, Welten M, Oddy W, Beilin L, Mori T, Jaddoe V, , Associations of maternal prepregnancy body mass index and gestational weight gain with cardio-metabolic risk factors in adolescent offspring: a prospective cohort study, BJOG 123 (2016) 207–216. 26525168 10.1111/1471-0528.13700

[48] Winkler AM, Ridgway GR, Webster MA, Smith SM, Nichols TE, Permutation inference for the general linear model, NeuroImage. 92 (2014) 381–397. 24530839 10.1016/j.neuroimage.2014.01.060 PMC4010955

[49] Jacob C, Statistical power analysis for the behavioral sciences, 2nd edition, Lawrence Erlbaum Associates, Hillsdale, NJ, 1988.

[50] Maugeri A, Barchitta M, Favara G, La Mastra C, La Rosa MC, Magnano San Lio R, , The application of clustering on principal components for nutritional epidemiology: a workflow to derive dietary patterns, Nutrients 15 (2022) 195. 36615850 10.3390/nu15010195 PMC9824338

[51] Bartenhagen C, Klein H-U, Ruckert C, Jiang X, Dugas M, Comparative study of unsupervised dimension reduction techniques for the visualization of microarray gene expression data, BMC Bioinform 11 (2010) 567. 10.1186/1471-2105-11-567 PMC2998530 21087509

[52] Henning C, Cluster-wise assessment of cluster stability, Comput. Stat. Data Anal. 52 (2007) 258–271. Available from: https://www.homepages.ucl.ac.uk/~ucakche/papers/clusta.pdf.

[53] Hsu D, Comparison of integrated clustering methods for accurate and stable prediction of building energy consumption data, Appl. Energy. 160 (2015) 153–163.

[54] Turk N, Continuous glucose monitoring in patients with postbariatric hypoglycemia: effect on hypoglycemia and quality of life, J. Endocr. Soc. 9 (9) (2025) bvaf106. 40862087 10.1210/jendso/bvaf106 PMC12371835

[55] Lasekan JB, Jacobs J, Reisinger KS, Montalto MB, Frantz MP, Blatter MM, Lactose-free milk protein-based infant formula: impact on growth and gastrointestinal tolerance in infants, Clin. Pediatr. 50 (2011) 330–337. 10.1177/0009922810390511 21436148

[56] Alsaleem M, Dusin J, Akangire G, Effect of low lactose formula on the short-term outcomes of neonatal abstinence syndrome: a systematic review, Glob. Pediatr. Health 8 (2021), 2333794X211035258. 10.1177/2333794X211035258 PMC8312188 34368403

[57] Goran MI, Descarpentrie A, Adise S, Factors that shape dietary intake in children in the context of increasing risk for obesity development, Pediatr. Obes. 20 (2025) e70004. 40050135 10.1111/ijpo.70004

[58] Goran MI, Plows JF, Ventura EE, Effects of consuming sugars and alternative sweeteners during pregnancy on maternal and child health: evidence for a secondhand sugar effect, Proc. Nutr. Soc. 78 (2019) 262–271. 30501650 10.1017/S002966511800263X PMC7441786

[59] Hill NR, Oliver NS, Choudhary P, Levy JC, Hindmarsh P, Matthews DR, Normal reference range for mean tissue glucose and glycemic variability derived from continuous glucose monitoring for subjects without diabetes in different ethnic groups, Diabetes Technol, Ther 13 (2011) 921–928. 21714681 10.1089/dia.2010.0247 PMC3160264

[60] Naguib MN, Hegedus E, Raymond JK, Goran MI, Salvy S-J, Wee CP, , Continuous glucose monitoring in adolescents with obesity: monitoring of glucose profiles, glycemic excursions, and adherence to time restricted eating programs, Front. Endocrinol. 13 (2022) 841838. 10.3389/fendo.2022.841838 PMC8914373 35282464

