# Supplementary material for: Carbohydrate composition of infant formula and glycemic regulation in early infancy using continuous glucose monitoring: cross-sectional evidence of altered glucose patterns with corn syrup solid-based formulas
Source: Am J Clin Nutr. Author manuscript; Available in PMC 2026 Jul 2. (PMC13327359; doi:10.1016/j.ajcnut.2026.101325)
Supplement: MMC1 [file NIHMS2177260-supplement-MMC1.docx]

**Supplemental Figure 1.** Overlaid histograms depicting the distribution of continuous glucose monitoring data, from all infants combined. Non-winsorized (red), winsorized (light blue), and overlapping (gray) values reveal no significant differences in distribution based on wilcoxon rank sum test (p > 0.05). Only values below 60 mg/dL were replaced with 60 mg/dL. All other values remained unchanged.

**Supplemental Figure 2.** Low blood glucose readings (compression lows; < 60 mg/dL), found in 22 infants, occur disproportionately more at nighttime (22:00 to 06:59) than daytime (07:00-21:59). (A) Histogram of glucose readings depicting the quantity of infants (y-axis) and the frequency of low blood glucose readings (x-axis). (B) Boxplots display the proportion of low glucose events per infant during daytime and nighttime. Each line tracks an individual infant’s data. Wilcoxon signed-rank test reveals a significant increase in nighttime low events for nearly all infants.

**Supplemental Figure 3.** Histogram depicting the distribution of CGM wear time (days) across all infants that passed data quality control. Blue lines depict mean (6.0; solid blue line) and standard deviation (1.2; dashed blue lines). Most infants contributed 6 full days of CGM data following data quality control.

**Supplemental Table 1.** Median [interquartile range] descriptive statistics, permutation-based Freedman–Lane ANCOVA results, and pairwise comparisons of CGM-derived metrics across infant feeding strategy. Pairwise comparisons were conducted within the same permutation-based resampling framework with Benjamini–Hochberg false discovery rate (FDR) correction applied across comparisons. Effect sizes for pairwise comparisons are reported as partial η²ₚ with 95% bootstrap confidence intervals (BCIs) derived from permutation resampling (B = 9,999). Bolded p-values indicate statistical significance (p < 0.05) and bolded effect sizes denote large effects (partial η²ₚ > 0.14), for both the overall Freedman–Lane ANCOVA and pairwise comparisons. All p-values were adjusted for multiple comparisons using the Benjamini–Hochberg procedure.


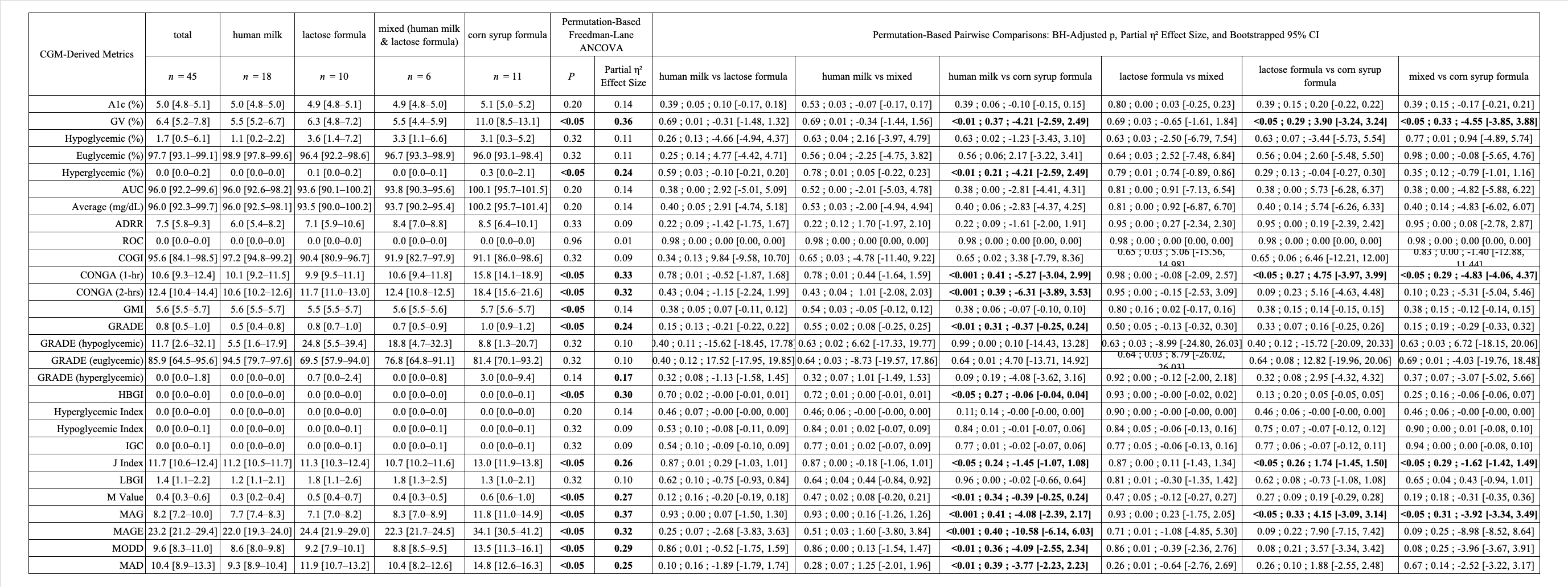


Abbreviations: A1c, hemoglobin A1c; GV, glucose variability; Hypoglycemic, < 70 mg/dL; Euglycemic, 71 – 139 mg/dL; Hyperglycemic, >140 mg/dL; AUC, area under curve; ADRR, average daily risk range; ROC, rate of change; COGI, continuous glucose monitoring index; CONGA, continuous overall net glycemic action; GMI, glucose management indicator; GRADE, glycemic risk assessment diabetes equation; HBGI, high blood glucose index, IGC; index of glycemic control; LBGI; low blood glucose index; MAG, mean absolute glucose; MAGE, mean amplitude of glycemic excursions; MODD, meann of daily differences; MAD, median absolute deviation.

**Supplemental Table 2.** Descriptive statistics of CGM data across all infants per feeding strategy.


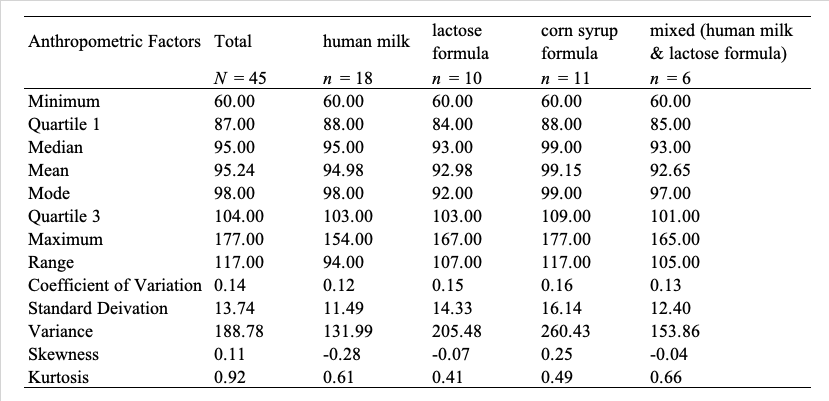


**Supplemental Table 3.** Comparison of infant /mother dyad anthropometric characteristics across feeding strategy based on categorical and continuous data.


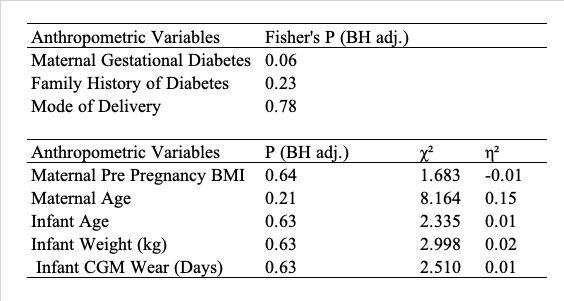

Abbreviations: χ² = chi square; η² = effect size

**Supplemental Table 4. M**edian [interquartile range] descriptive statistics, Kruskal-Wallis test results, and pairwise comparisons of dietary variables across infant feeding strategy. Pairwise comparisons were conducted within the same permutation-based resampling framework with Benjamini–Hochberg false discovery rate (FDR) correction applied across comparisons. Effect sizes for pairwise comparisons are reported as partial η²ₚ with 95% bootstrap confidence intervals (BCIs) derived from permutation resampling (B = 9,999). Chi-square values are reported for categorical variables. Bolded p-values indicate statistical significance (p < 0.05) and bolded effect sizes denote large effects (η² > 0.14 for Kruskal-Wallis; partial η²ₚ > 0.14 for pairwise comparisons), for both the overall Kruskal-Wallis tests and pairwise comparisons. All p-values were adjusted for multiple comparisons using the Benjamini–Hochberg procedure.

**
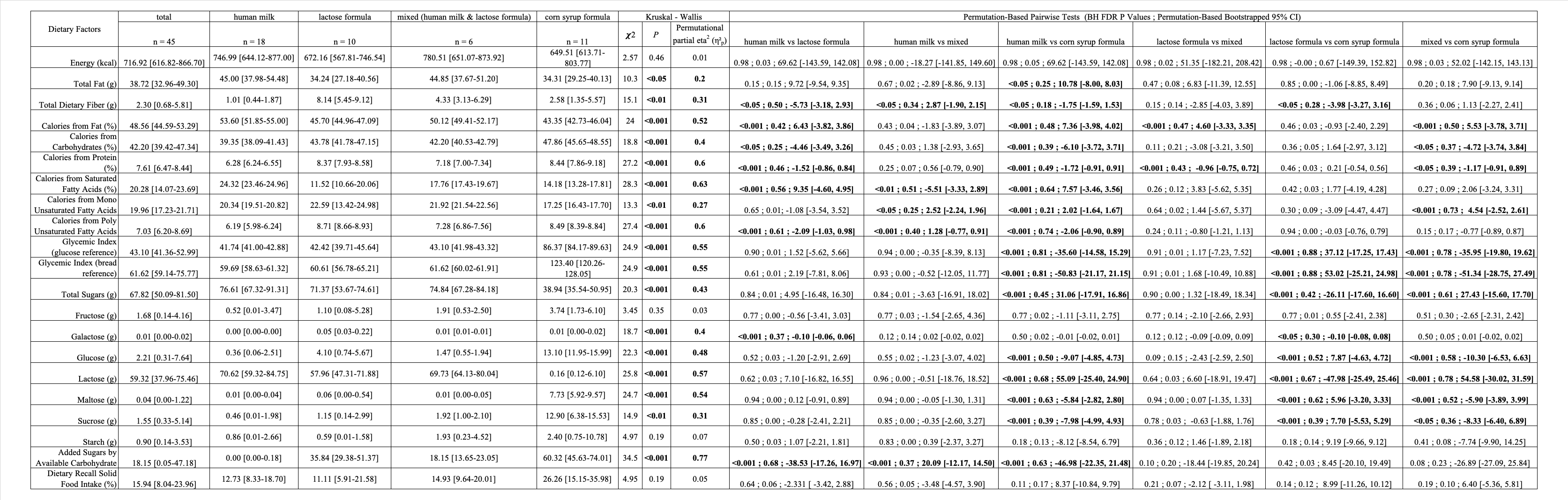
**

**Supplemental Figure 4.** Pearson correlation of 28-CGM derived metrics clustered by similarities in correlation patterns. Blue panels indicate correlations > 0, red panels indicated correlations < 0, and white represents no correlation.

**Supplemental Figure 5.** Principal component analysis of 28 CGM-derived metrics from all infants reveals that components 1 and 2 explain 44.2% and 38.6% of variance respectively, totaling > 80% cumulative variance (dashed horizontal blue line). PC1 and PC2 were used for subsequent hierarchical clustering.

**Supplemental Figure 6.** Hierarchical classification reveals four distinct clusters. (A) Wards D2 hierarchical clustering of principal components reveals four clusters. (B,C) Silhouette width and elbow plot reveal 4 distinct clusters. (D) Jaccard stability and bootstrapping indicate strong stability at 4 clusters.

**Supplemental Figure 7.** Grid of individual infant longitudinal CGM data sorted based on infant glycemic clusters labelled in headers. Each plot represents one infant. Hypoglycemic (< 70 mg/dL; purple) and hyperglycemic (> 140 mg/dL; orange) incidents are depicted by highlighted points.

**Supplemental Table 5.** Descriptive statistics, multivariate analysis, and pair-wise comparisons of CGM-derived metrics across glycemic clusters.


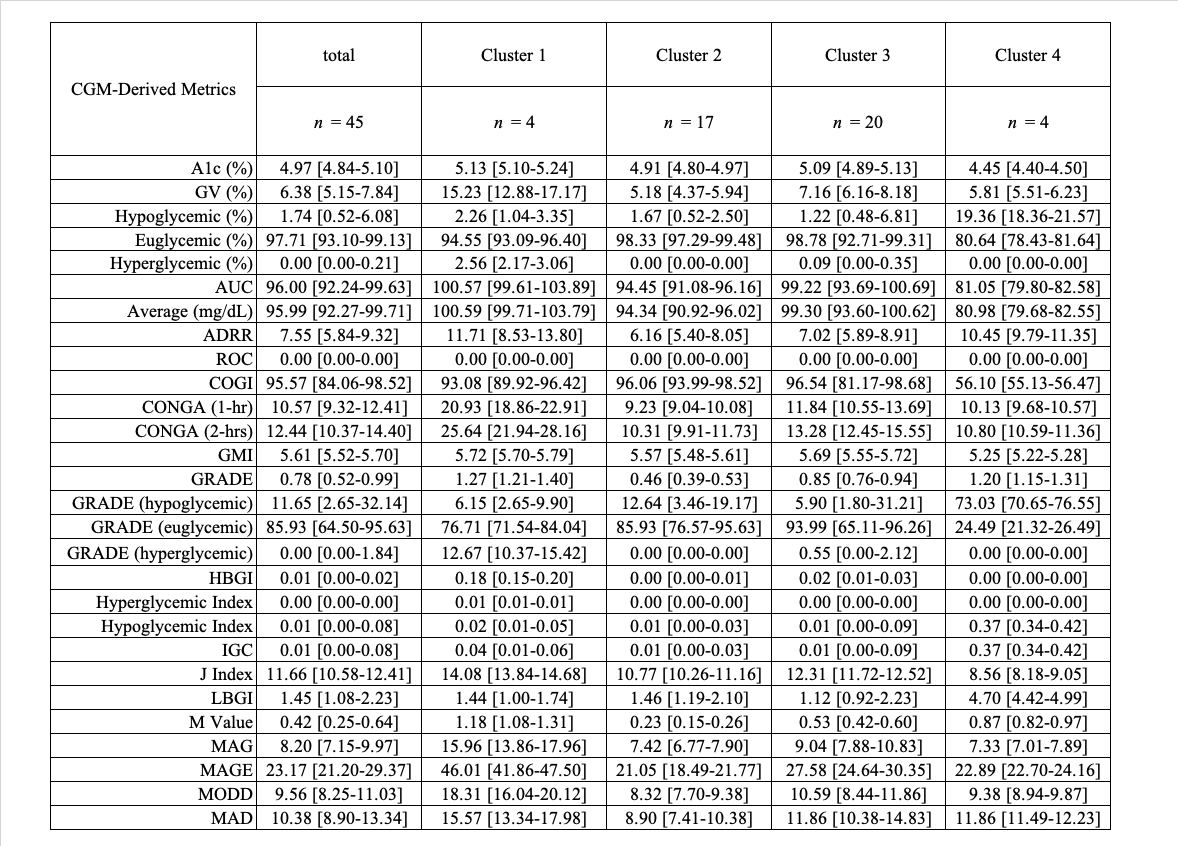


Abbreviations: A1c, hemoglobin A1c; GV, glucose variability; Hypoglycemic, < 70 mg/dL; Euglycemic, 71 – 139 mg/dL; Hyperglycemic, >140 mg/dL; AUC, area under curve; ADRR, average daily risk range; ROC, rate of change; COGI, continuous glucose monitoring index; CONGA, continuous overall net glycemic action; GMI, glucose management indicator; GRADE, glycemic risk assessment diabetes equation; HBGI, high blood glucose index, IGC; index of glycemic control; LBGI; low blood glucose index; MAG, mean absolute glucose; MAGE, mean amplitude of glycemic excursions; MODD, mean of daily differences; MAD, median absolute deviation.

**Supplemental Table 6.** Select dietary characteristics of infant feeding strategies based on

recalls.


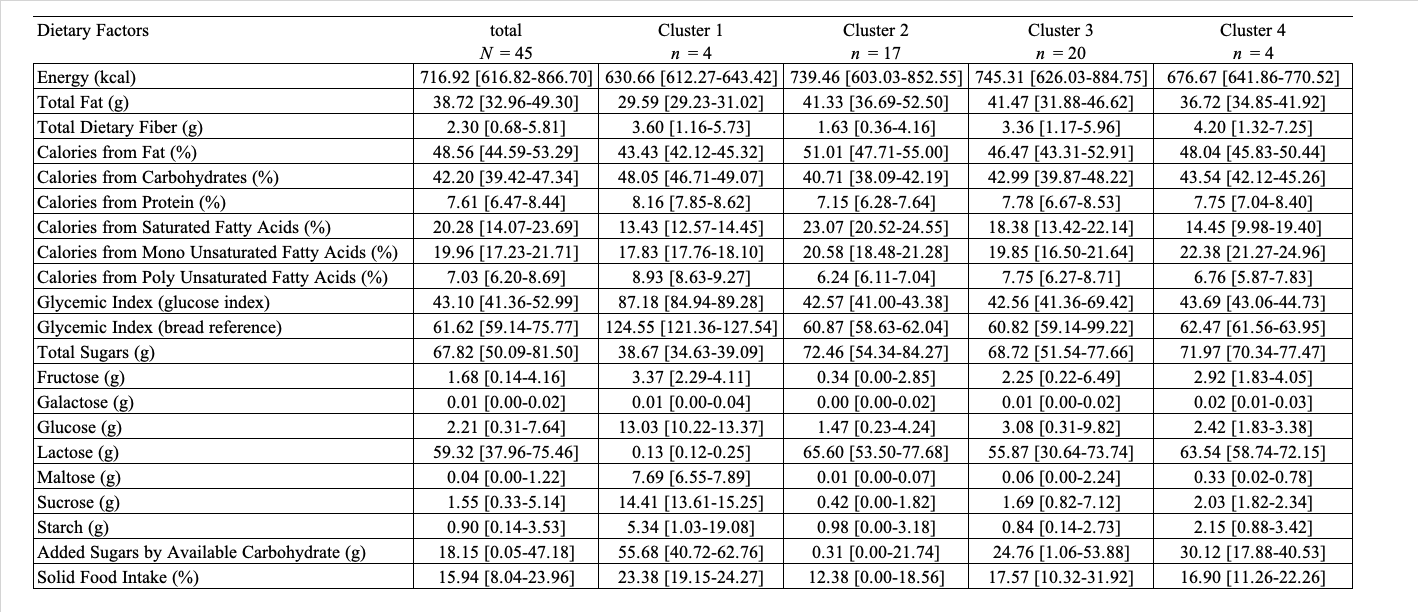


Factors are expressed as mean (SD) for dietary variables. Superscripted letters denote significant differences across pair-wise comparisons via Dunn’s post hoc test and Benjamini-Hochberg FDR correction.
